# A Multi-Region Magnetoimpedance-Based Bio-Analytical System for Ultrasensitive Simultaneous Determination of Cardiac Biomarkers Myoglobin and C-Reactive Protein

**DOI:** 10.3390/s18061765

**Published:** 2018-06-01

**Authors:** Zhen Yang, Huanhuan Wang, Pengfei Guo, Yuanyuan Ding, Chong Lei, Yongsong Luo

**Affiliations:** 1 School of Physics and Electronic Engineering, Xinyang Normal University, Xinyang 464000, China; 13323978535@163.com (H.W.); guopengfei2010@126.com (P.G.); m15037680221@16.com (Y.D.); eysluo@163.com (Y.L.); 2 Key Laboratory of Microelectronics and Energy of Henan Province, Xinyang Normal University, Xinyang 464000, China; 3 Department of Micro/Nano Electronics, School of electronic information and electrical engineering, Shanghai Jiao Tong University, Dongchuan Road 800, Shanghai 200240, China; leiqhd@sjtu.edu.cn

**Keywords:** myoglobin, C-reactive protein, Dynabeads, magnetoimpedance, microfluidic device

## Abstract

Cardiac biomarkers (CBs) are substances that appear in the blood when the heart is damaged or stressed. Measurements of the level of CBs can be used in course of diagnostics or monitoring the state of the health of group risk persons. A multi-region bio-analytical system (MRBAS) based on magnetoimpedance (MI) changes was proposed for ultrasensitive simultaneous detection of CBs myoglobin (Mb) and C-reactive protein (CRP). The microfluidic device was designed and developed using standard microfabrication techniques for their usage in different regions, which were pre-modified with specific antibody for specified detection. Mb and CRP antigens labels attached to commercial Dynabeads with selected concentrations were trapped in different detection regions. The MI response of the triple sensitive element was carefully evaluated in initial state and in the presence of biomarkers. The results showed that the MI-based bio-sensing system had high selectivity and sensitivity for detection of CBs. Compared with the control region, ultrasensitive detections of CRP and Mb were accomplished with the detection limits of 1.0 pg/mL and 0.1 pg/mL, respectively. The linear detection range contained low concentration detection area and high concentration detection area, which were 1 pg/mL–10 ng/mL, 10–100 ng/mL for CRP, and 0.1 pg/mL–1 ng/mL, 1 n/mL–80 ng/mL for Mb. The measurement technique presented here provides a new methodology for multi-target biomolecules rapid testing.

## 1. Introduction

Measurements of the level of cardiac biomarkers (CBs) is important when the heart is damaged or stressed, especially in the case of cardiovascular disease (CVD) risk [1,2]. Among various kinds of cardiac biomarkers, C-reactive protein (CRP) is a most important biomarker for CVD risk. The level of CRP can rise from a normal level of less than 5 mg/L to above 100 mg/L after acute inflammatory stimulus [3]. Myoglobin (Mb) is one of the early biomarker levels which increases sharply from 90 pg/mL to 250 ng/mL within 90 min after acute myocardial infarction (AMI), playing a major role in urgent diagnosis of CVD [4,5,6]. More and more researchers have attempted to apply different types of biosensors to detect the levels of two biomarkers [7,8,9,10,11]. Fluorescence-linked immunosorbent assay was widely used for detection of CBs; however, it is frequently used in in vitro diagnosis and complicated operation steps limit its application [12,13]. Immunosensors based on electrochemical impedance spectroscopy (EIS) usually use an immobilized recognition element (probe) to bind the target/analyte molecule selectively. However, the surface of the biosensors suffer from perturbations on the sensor surface that are influenced by different pH, ionic strength and co-existing molecules in biological fluids. So, the sensitivity, specificity, and relevant range are affected [14,15]. Magnetic bio-detection based on giant magnetoimpedance was proposed long ago [16,17]. Magnetoimpedance (MI) is the change of total impedance of ferromagnetic-conducting sensitive elements under application of an external magnetic field [18,19,20]. Recently, different MI-based bio-sensing systems for micro-sized/nano-sized magnetic particles and single biomarker detection were designed and tested [21,22,23,24,25,26,27,28].

Despite the progress in medical diagnostics, CVD is considered to be the most common disease which occurs in middle age and elderly people, especially those over 50 years old [29]. The symptoms are complex and associated with more than one biomarker. Detection of a single cardiac biomarker is usually not sufficient for precise CVD diagnostics, due to the limited specificity [30]. Therefore, there is an urgent need for development of a simple, rapid, highly sensitive and inexpensive system for simultaneous detection of several cardiac biomarkers. The advantage of a microfluidic device (MFD) is that different biomarkers can be tested in various detection regions pre-modified with different specified antibodies, i.e. the development of multi-analyte immunoassay combined with a multi-region bio-analytical system (MRBAS) is a very important task [31,32,33,34,35]. The studies in recent years indicated that a higher MI effect can be reached in a multilayered structure and in thin films shaped as meanders [36,37,38,39,40]. Both high MI response and high sensitivity with respect to an applied magnetic field are the main factors for MI bio-sensing applications.

In this paper, we designed and tested the ultrasensitive bio-analytical MI-based system for simultaneous detection of CRP and Mb, based on optimization of the structural parameters of the MI element. Immune reaction was performed in the MRBAS based on MI changes. The MI response of the sensitive element was measured in its initial state and in the presence of biomarkers. Compared with the control region, ultrasensitive and combined detection of CRP and Mb was accomplished in the different detection regions. The methodology presented here provides a vital basis for multi-analyte bio-magnetic detection like Troponin, Creatine Kinase-MB, alpha-fetoprotein (AFP) and carcinoembryonic antigen (CEA).

## 2. Materials and Methods

### 2.1. Reagents and Instruments

All the biological and chemical reagents used for the present study are listed in Table 1. For all experiments described here, deionized water was used. The MI multilayered sensitive element and Multi-region microfluidic devices (MR-MFD) were prepared by widely employed micro electromechanical system technology (MEMS) in National Key Laboratory (China). MI measurements were made using a special system based on a Hewlett-Packard 4194A Impedance analyzer (Agilent Technologies Inc., Palo Alto, CA, USA). The programmable syringe pump (PHD 4400 HPSI, Harvard Apparatus, Holliston, MA, USA) was connected with the MFD and adopted for injecting test samples. All measurements were made at room temperature.

### 2.2. Microfabrication of MI Element and MR-MFD

**Figure 1:**
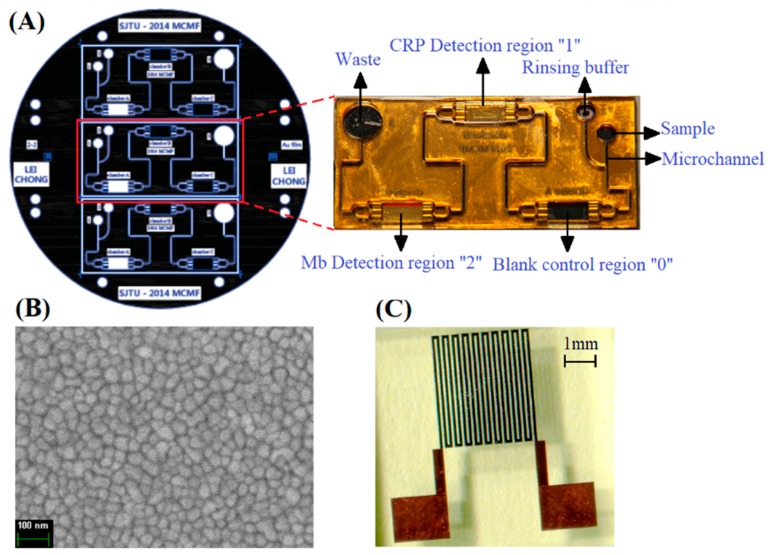
(**A**) The designed diagram of the microfluidic device, and the inset showing the photograph of the fabricated microfluidic device. (**B**) The Scanning Electron Microscopy image of the thin gold film. (**C**) The fabricated multilayer MI element.

The meander-shaped MI element was designed for superior structural parameters, and the manufacturing process was presented in Figure S1, following our previous experiments described elsewhere [41]. The electrodeposited NiFe layer had good soft magnetic properties, which were similar to those previously studied in in our works [42,43]. Figure 1A shows the design diagram of MR-MFD. Two detection regions (CRP detection region 1 & Mb detection region 2), one blank control region and two inlets (sample and region buffer) were designed. The distance between any of two adjacent detection regions are 20 mm in order to minimize the magnetic interference between them. Each detection region contained a single rectangular gold film unit and possessed an area of 3 × 5 mm^2^, and the control region possessed the same area without the gold film unit. The MR-MFD were fabricated based on SU-8 and polydimethylsiloxane (PDMS) materials by MEMS technology. The general view of the fabricated MI element is shown in Figure 1C. The MR-MFD containing the fluid reservoir and microfluidic pipeline were designed for sandwich immunoassay. Following is the step-by-step description of MR-MFD preparation:

1. Fabrication of gold layer: First a chromium adhesion layer (~60 nm) was deposited on the glass wafer with a thickness of 1 mm at a rate of 1 Å s^−1^, followed by ~240 nm of gold at a rate of 2–3 Å s^−1^ by a radio frequency sputtering system (LH-Z550, Shanghai, China).
2. Patterning of the gold film: A photoresist layer with a thickness of 10 μm was spun onto the Au layer and afterwards patterned to several small MFD units through the mask.
3. Deleting the uncovered part of the gold layer: the uncovered part of the Au layer was removed by wet etching in the KI, I_2_ and H_2_O mixed solution for 45 s.
4. Deleting the photoresist: The whole glass substrate with Au film was immersed in acetone solution for 25 s.
5. SU-8 layers preparation: The SU-8 photoresist with a thickness of 500 μm was spin coated on Au film, soft baked, patterned with a mask, and developed with an SU-8 developer. Then, the same thickness of the SU-8 photoresist was spun again, finally a 5 × 3 mm^2^ rectangular microcavity with a depth of 1 mm was achieved. Figure 1B showed the SEM of gold nanofilm.
6. PDMS casting: The pre-polymer and the curing agent of PDMS were mixed in a 10:1 ratio by weight. After thermal coagulation, the PDMS can be obtained.
7. Bonding of the SU-8 with the PDMS: The surface-treated PDMS was tightly bound to the Su-8 surface and then opened the inlets and waste chamber. Figure 1A (inset) shows the fabricated microfluidic device without PDMS.

### 2.3. Sandwich Immunoassay for Capturing CBs

**Figure 2:**
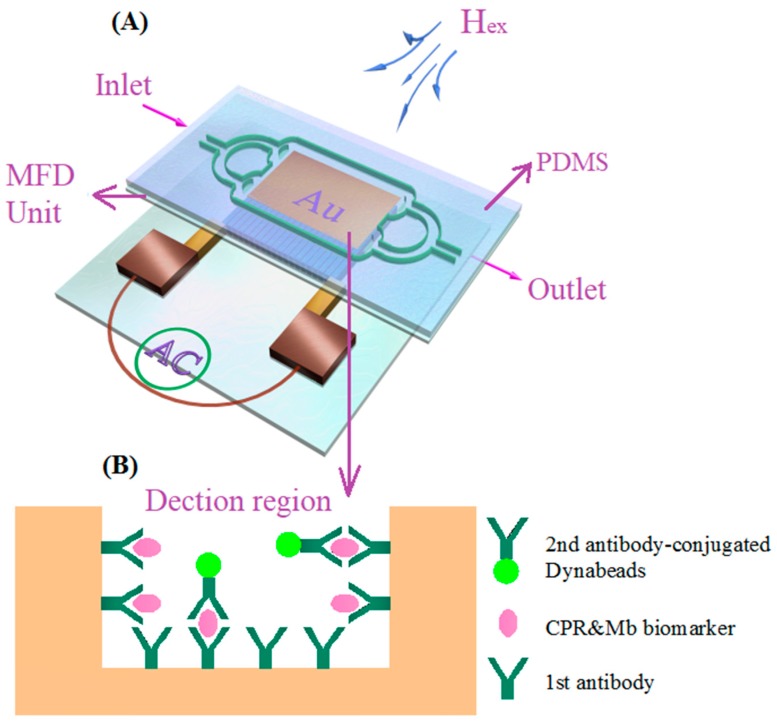
The principle diagram of detection for cardiac biomarkers using an MI sensitive element in the shape of the meander (**A**); general illustration of the biochemical part of the detection part (**B**).

The detection regions were pre-modified through self-assembly and activation, prior to PDMS bonding. To achieve selectivity and improve the efficiency of detection, the detection regions 1 and 2 were surface modified with a mouse CRP monoclonal antibody or mouse Mb monoclonal antibody, respectively. The details for the self-assembling, activation and modification process can be found elsewhere [27]. After the surface modification, the detection regions were carefully rinsed with phosphate-buffered solution (PBS) prepared with deionized water. Afterwards, the detection regions were sealed with 100 μL BSA solution (including 1% BSA, 0.2% tween 20) at 4 °C for 2 h and washed twice with PBS solution (PH = 7.4). The CRP and Mb antigen complexes with different concentrations (1 pg/mL–100 ng/mL for CRP and 0.1 pg/mL–80 ng/mL for Mb) were placed into the MR-MFD by a simple syringe pump. The complexes flowed through different regions in turn. Immunoassay time was as long as 20 min, aiming to ensure an effective combination of antigen-antibody. Finally, The PBS solution was used for rinsing. Then, biotinylated CRP & Mb polyclonal antibody (10 μL, 1 mg/mL) mixed solution was injected into the MR-MFD and washed five times. Finally, Dynabeads suspension (40 μL, 10 μg/mL) was injected. Dynabeads are polystyrene/iron oxide nanomaterials (superparamagnetic spheres) widely used for magnetic separation and biosensing [44,45,46]. After cultivating, washing and drying the whole double antibody immunoassay process was completed as shown in Figure 2B.

### 2.4. Determination of CBs Using MI-Based Bio-Analytic System

The CRP and Mb antigen were mixed with the same concentration and volume (10 μL) in all cases under consideration. Different detection regions modified by different antibodies would capture different CBs. First of all, the MI response of the thin film element located below the blank control region was measured in its initial state. The MI response of the thin film element located below the detection regions 1and 2 were measured in the presence of CBs. The fundamental principle for detection of Dynabead-labeled CBs is the detection of the stray fields (H_stray_) of the Dynabeads. This is a similar principle as it was previously discussed in many cases of different magnetic biosensors [17,37,38,39]. When the MI element was moved to the bottom of the detection region of MR-MFD, the Dynabeads trapped in the MFD unit, located above the MI element as shown in Figure 2A, were magnetized by an external magnetic field (H_ex_: 0–120 Oe) created by a pair of Helmholtz coils and they emitted a detectable H_stray_. The H_stray_ modified the partially overlapping magnetic field near the MI element and resulted in changes of transverse permeability and skin penetration depth; this lead to the altering of MI. The different concentrations of CBs flowing through the MR-MFD corresponds to the changes of MI. The advantages of this MI-based MRBAS is that the different MI responses in the varying regions reflect the content for CRP and Mb, simultaneously. The effect of H_stray_ on MI is affected by the distance of the MR-MFD to the MI element; therefore, the thinnest glass wafer is selected to use as a substrate for MR-MFD fabrication. The MI ratios were calculated as follows:

(1) $$MI\;ratio=\frac{\Delta Z}{Z}=100\times \frac{Z(H)-Z(H_{\max})}{Z(H_{\max})}$$

where *H*_max_ = 120 Oe for orientation of the external magnetic field along the long side of the meander thin film elements.

## 3. Results and Discussion

The maximum MI sensitivity of the fabricated sensitive element of about 22% Oe^−1^ was obtained at 1.4 MHz and 8.8 Oe field. Different antigen molecules were captured in different detection regions. The antigen molecules were combined with a certain number of magnetic beads. The beads captured in different detection regions were magnetized in varying degrees and behaved as a magnetic dipole producing stray fields H_stray_, disturbing the external magnetic field. Therefore, the original transverse permeability μ_T_ of the MI sensitive element experiences a different resultant magnetic field H_R_ = H_ex_ + h_AC_ + H_stray_ (h_AC_ was AC current magnetic field) and achieves a different value of field superposition giving rise to a different value in the MI ratio. The MI response quantitatively reflects the presence, content, or the absence of biomarkers. 

**Figure 3:**
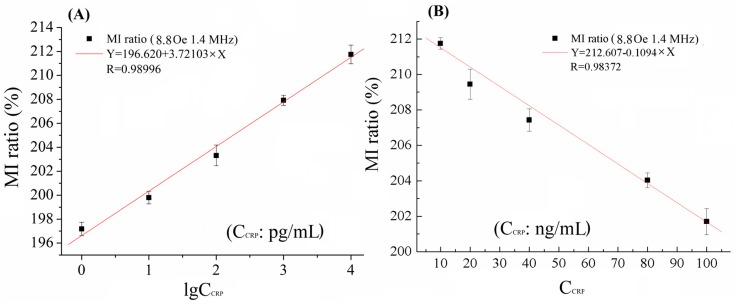
The relationship of MI ratio versus (**A**) low CRP concentrations and (**B**) high CRP concentrations at f = 1.4 MHz and H_ex_ = 8.8 Oe.

Figure 3 shows the relationship between the MI responses and CRP concentrations (C_CRP_). The field dependences of the MI ratio for detecting different concentration of CRP are shown in Figure 2 and Figure 3. Evidently, the MI ratios have been enhanced by distinct values on account of the CRP antigen conjugated with magnetic beads with different concentrations in the detection region 1, from 1 pg/mL to 10 ng/mL. It was worthwhile to note that the MI ratio increased from 195% (without CRP antigen) to 212% with increasing the concentration of the CRP antigen. For each concentration, independent measurement was performed 10 times under the same testing conditions. The relative standard deviation (RSD) is reasonable, as can be seen in Figure 3A; this indicates good precision.

The linearity between the MI response signals and CRP concentrations with the range of 1 pg/mL–10 ng/mL, is represented under 8.8 Oe and 1.4 MHz by the fitting form in base-10 logarithms. As can be seen clearly from Figure 3A, 5 points locate near the curve and R = 0.98996 is very close to 1; we can say that the MI response has a good linear relationship with the CRP concentrations. However, the MI ratio began to fall down from 218 to 202% with the change of concentration from 10 ng/mL to 100 ng/mL, as can be seen in Figure S3. The linearity was also tested in this concentration range, displaying a statistical curve fitted by the linear regression. Five points locate near the curve and R = 0.98372 is very close to 1, as shown in Figure 3B. Therefore, lower and upper limitations of detection were 1 pg/mL and 100 ng/mL, respectively.

**Figure 4:**
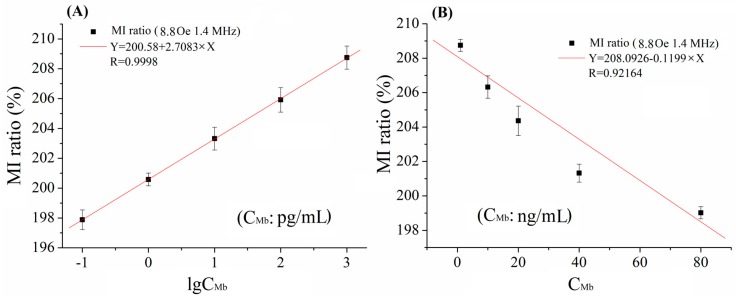
The relationship of MI ratio versus (**A**) low Mb concentrations and (**B**) high Mb concentrations at f = 1.4 MHz and H_ex_ = 8.8 Oe.

In our previous work [25], we found that Dynabeads (conjugated 10 ng/mL biomarkers) on the surface of the element were nearly magnetically saturated. The high concentration of Dynabeads had caused the high-density clusters of Dynabeads, the interaction between beads was obvious, the whole H_stray_ attenuated, and the added value of the MI was reduced. A similar result on Mb concentration (C_Mb_) detection was obtained in the detection region 2, as shown in Figure 4. However, the linear detection ranges were 0.1 pg/mL–1 ng/mL and 1 n/mL–80 ng/mL for Mb. From Figure 3 and Figure 4, we observed the overlap MI value in low concentration and high concentration for CRP and Mb. Therefore, dual-measurement for the same CBs sample would be better by using the designed MI-based bio-sensing system. Firstly, we can measure the sample with original concentration, and then measure the sample with decreased concentration by mixing with a buffer (e.g., in a ratio of 1:1). Then the region of concentration of the sample can be pinpointed depending on the trend of the MI ratio.

**Figure 5:**
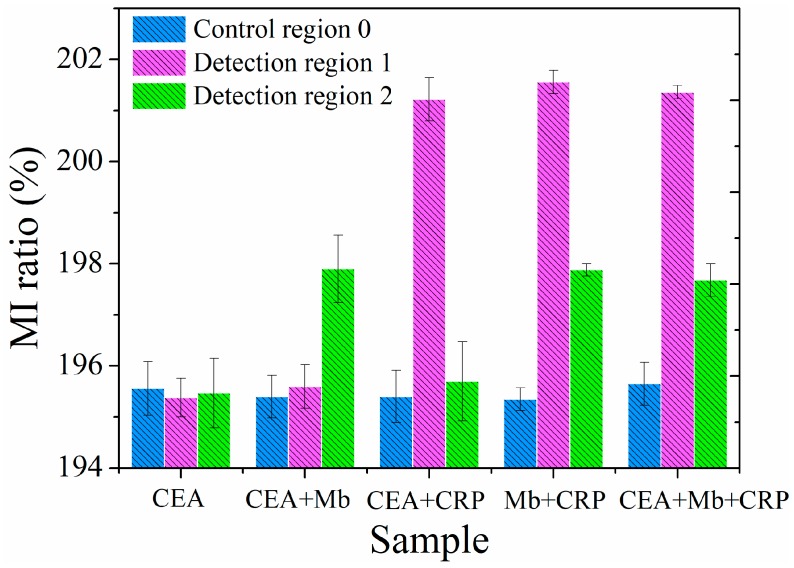
MI responses for the specificity test on CRP and Mb; single CEA antigen solution and four antigens mixture solution (CEA + Mb; CEA + CRP; CRP + Mb, CEA + Mb + CRP) were injected into the MR-MFD in turn.

**Figure 6:**
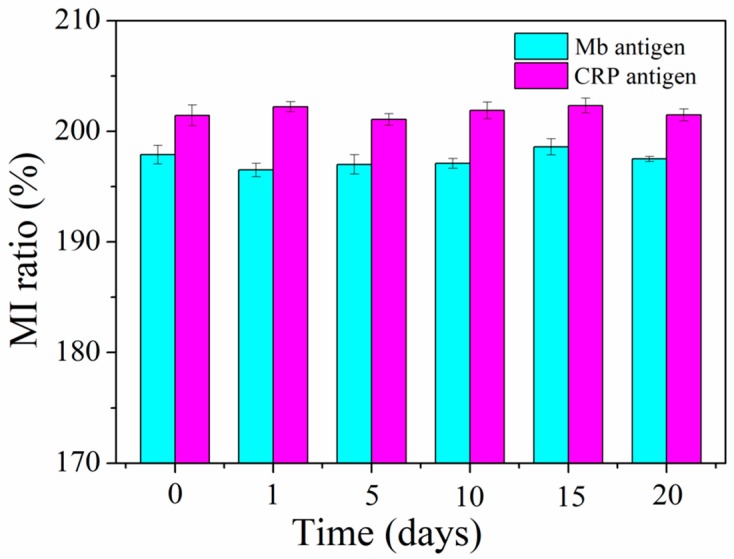
Stability test of the MI-based bio-analytic system in 0.1 pg/mL Mb and 100 ng/mL CRP.

Sensitivity and specificity are two major factors to assess the practicality of immunosensors. In addition to the above sensitivity analysis, the specificity test of the MI-based bio-analytical system was performed by interfering antigen CEA. The single interfering antigen solution (0.1 pg/mL CEA) and four mixture antigen solutions (0.1 pg/mL CEA + 0.1 pg/mL Mb, 0.1 pg/mL CEA + 100 ng/mL CRP, 0.1 pg/mL Mb + 100 ng/mL CRP and 0.1 pg/mL CEA + 0.1 pg/mL Mb + 100 ng/mL CRP) were infused into the MR-MFD by syringe pump, respectively. The MI response was observed in controlled region 0 and detection regions 1 and 2, with a specific antigen-antibody reaction. The MI responses of the multilayered element under varying regions are shown in Figure 5. When 0.1 pg/mL CEA antigen solution was injected into the MR-MFD, there was no sharp difference for the MI ratio between control region 0 and detection region 1&2. An exclusive significant increase in the MI ratio was discovered in detection region 2 (with injecting the CEA + Mb antigens mixture solution) or in the detection region 1 (with injecting the CEA + CRP antigens mixture solution). However, significant variation of MI appeared simultaneously in detection region 1 and detection region 2 for CRP + Mb and CEA + Mb + CRP. The results indicated the selective binding of CRP in detection region 1 to the CRP antibody and Mb in detection region 2 to the Mb antibody. Thus, it illustrates the good specificity of the MI-based magnetic immunoassay for the detection of Mb and CRP. In view of the results of detection limit, we infused the mixed antigen (0.1 pg/mL CRP + 0.1 pg/mL Mb or 100 ng/mL CRP + 100 ng/mL Mb) into the MR-MFD, the same result was almost obtained like that in Figure 5 (Mb single antigen and CRP single antigen). So, in theory the detection limit for biomarkers is logical. The relative standard deviation (RSD) of 0.34% is obtained by performing 6 independent measurements (one time every ten minutes) on 0.1 pg/mL Mb under the same testing conditions as shown in Figure 6, indicating an acceptable reproducibility of the magnetic immunoassay. The stability capability test was repeated in different time (The corresponding time is 1st, 5th, 10th, 15th, 20th days, respectively). The high stability of the MI sensor contributes to the reliability of measurement results. The same reproducibility and stability test was performed on 100 ng/mL CRP. The RSD of the 6 independent assay results corresponded approximately to 0.648% and indicated the satisfactory reproducibility of the presented method. The results of the stability capability test confirm the stability of the MI element.

The detection limit of 1 ng/mL for CRP and 0.5 ng/mL for Mb was achieved by using an integrated ribbon-based MI biosensing system in our previous work [47,48]. In this work, the fabricated multilayered thin film MI sensitive element possesses a lower minimum detectable concentration of 1 pg/mL for CRP detection and 0.1 pg/mL for the Mb detection case. This can be attributed to the higher MI sensitivity of the multilayered thin film element. Although a lower detection limit of 0.029 pg/mL for CRP can be reached by applying a NH_2_-Ni-MOF electrocatalysts method [49] and detection limit is 0.01 pg/mL for Mb using an Au-WS_2_ nanohybrid based SERS aptasensor [50], utilization of the MRBAS results in ultrasensitive combined detection of CBs, Mb and CRP. Bio-magnetic detection has recently become a focus of interest for researchers because of its high sensitivity, versatile diagnostic methods, convenient processes, high accuracy and low cost. The detection limit with 0.01 pg/mL was considered more adequate for the quantification of Mb in clinical diagnostics. So, we will attempt to design more complex MR-MFD for ultrasensitive simultaneous detection of two, three or four biomarkers using the bio-magnetic measurement system in the future. Firstly, we are required to fabricate the higher sensitive MI element and MI element array by MEMS technology. Then, patterned microfluidic channel and magnetic nanoparticles labels will be considered for detection of CBs. Finally, the integrated MI-based biosensing system may be adopted.

## 4. Conclusions

In summary, ultrasensitive simultaneous detection of cardiac biomarkers Mb and CRP was achieved by the design and development of the MR MI-based bio-analytic system. The magnetic labels and double antibody sandwiched immunoassays were used for the measurements. The multilayered thin film MI element and MFD were manufactured by microfabrication techniques. The lower detection limits with 1 pg/mL for CRP and 0.1 pg/mL for Mb were obtained. The linear detection range contained a low concentration detection area and a high concentration detection area, which were 1 pg/mL–10 ng/mL, 10–100 ng/mL for CRP, and 0.1 pg/mL–1 ng/mL, 1 n/mL–80 ng/mL for Mb, respectively. The methodology presented here is promising for multi-analyte bio-magnetic detection of biocomponents (Troponin, CK-MB, AFP, CEA) and other biomarkers using MI-based biosensing system. 

## Figures and Tables

**Table 1:** Biological and chemical reagents.

Solution	Detail	Company
Mercaptopropionic acid	Concentration 20 mmol/L	Aladdin Chemistry Co. Ltd (Beijing, China)
EDC	Concentration 0.2 mol/L	Aladdin Chemistry Co. Ltd (Beijingi, China).
NHS	Concentration 0.05 mol/L	Shanghai Medpep Co. Ltd (Shanghai, China)
Mouse Mb &CRP 1st antibody	Concentration 1 mg/mL	Linc-Bio Science Co. Ltd. (Shanghai, China)
BSA	1% BSA, 0.2% tween 20	Via-gene pro bio Technologies Co. Ltd. (Shanghai, China)
Human Mb &CRP antigen	0.1–100 pg/mL, 1–100 ng/mL	Linc-Bio Science Co. Ltd. (Shanghai, China)
Mb &CRP 2 nd antibody	Concentration 1 mg/mL	Linc-Bio Science Co. Ltd. (Shanghai, China)
Dynabeads^®^ C1	Concentration 10 μg/mL	Invitrogen Co. Ltd. (Shanghai, China)
NaOH	Concentration 1 mol/L	Pinghu Chemical Reagent (Pinghu, China)
HCL	Concentration 1 mol/L	Sinpharm Chemical Reagent Co. Ltd. (Shanghai, China)
PBS	PH = 7.4	Medicago AB (Uppsala, Sweden)
C_3_H_6_O C_2_H_5_OH	AR	LingFeng Chemical Reagent Co. Ltd. (Shanghai, China)

## Supplementary Materials

The following are available online at http://www.mdpi.com/1424-8220/18/6/1765/s1, Figure S1: Fabrication steps of the MI element, Figure S2: Field dependence of MI responses under the low concentration of CRP antigen (a) full view (b) partial enlargement, Figure S3: Field dependence of MI responses under the high concentration of CRP antigen (a) full view (b) partial enlargement, Figure S4: SEM characterizations CRP-conjugated Dynabeads at the concentration of 10 ng/mL (A) and SEM characterizations Mb-conjugated Dynabeads at the concentration of 10 ng/mL (B).
