# Supplementary material for: A Multi-Region Magnetoimpedance-Based Bio-Analytical System for Ultrasensitive Simultaneous Determination of Cardiac Biomarkers Myoglobin and C-Reactive Protein
Source: Sensors (Basel). 2018 Jun 1;18(6):1765. doi: 10.3390/s18061765 (PMC6022111; doi:10.3390/s18061765)
Supplement: Supplementary file 1 [file sensors-18-01765-s001.pdf]

Supplementary Information

A Multi-Region Magnetoimpedance-Based Bio-Analytical System for Ultrasensitive Simultaneous Determination of Cardiac Biomarkers Myoglobin and C-reactive Protein

Zhen Yang [1,2,*], Huanhuan Wang [1,2], Pengfei Guo [1,2], Yuanyuan Ding [1,2] Chong Lei [3] and Yongsong Luo [1,2]

[1] School of Physics and Electronic Engineering, Xinyang Normal University, Xinyang 464000, China; 13323978535@163.com (H.W.); guopengfei2010@126.com (P.G.); m15037680221@16.com (Y.D); eysluo@163.com (Y.L.)

[2] Key Laboratory of Microelectronics and Energy of Henan Province, Xinyang Normal University, Xinyang 464000, China;

[3] Department of Micro/Nano Electronics, School of electronic information and electrical engineering, Shanghai Jiao Tong University, Dongchuan Road 800, Shanghai 200240, China; leiqhd@sjtu.edu.cn

2.2. Microfabrication of MI element and MR-MFD

The meander-shaped MI element was designed for appropriate structural parameters in order to obtain the high performance in this work. The length of the straight-line segment in the tortuous structure was 5 mm. The widths of NiFe and Cu film were 160 and 140 μm, respectively. The space between neighboring segments was 80 μm. The thicknesses of the NiFe and Cu film were about 3 and 2 μm, respectively.

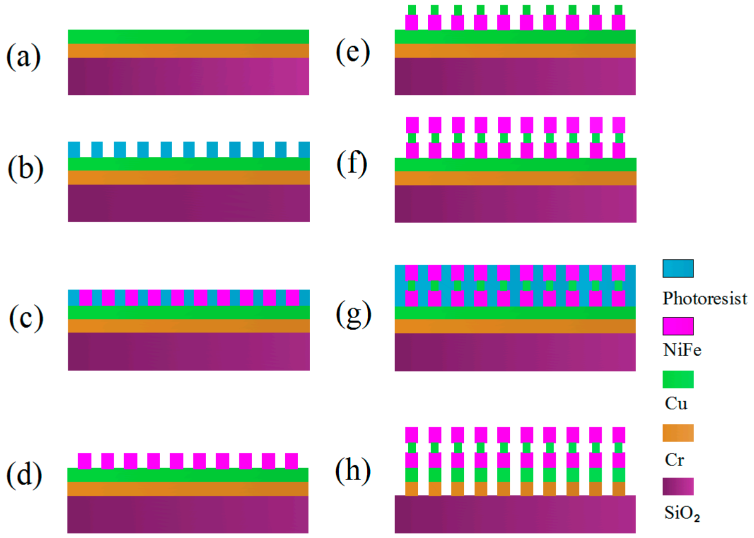

Figure S1. Fabrication steps of the MI element.

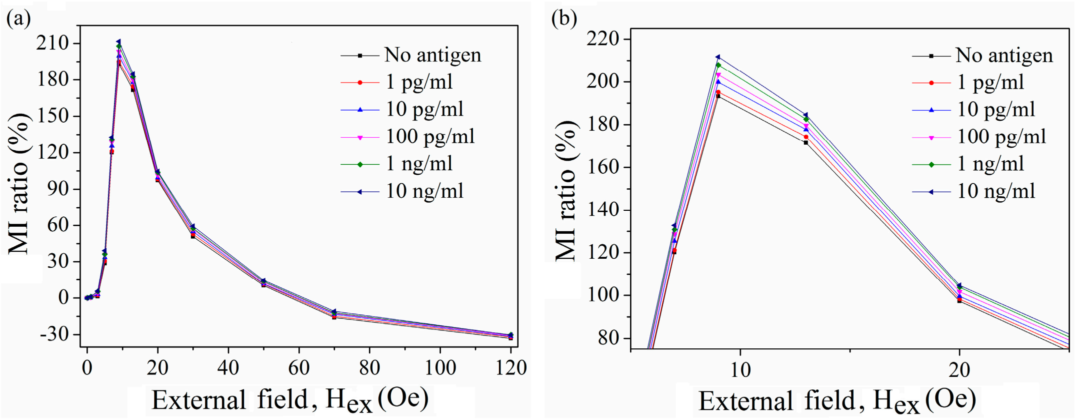

Figure S2. Field dependence of MI responses under the low concentration of CRP antigen (a) full view (b) partial enlargement.

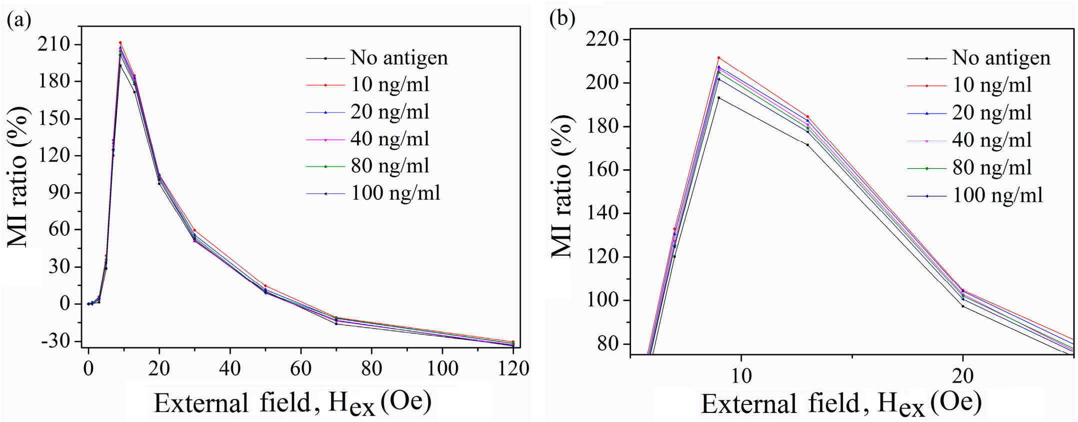

Figure S3. Field dependence of MI responses under the high concentration of CRP antigen (a) full view (b) partial enlargement.

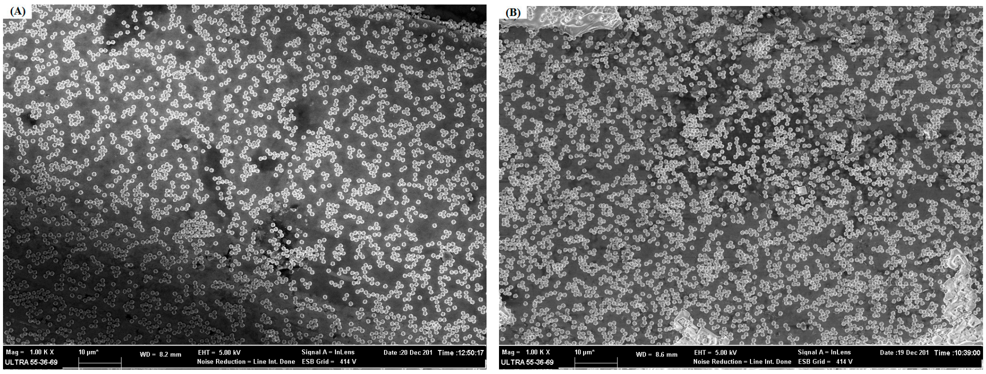

Figure S4. SEM characterizations CRP-conjugated Dynabeads at the concentration of 10 ng/ml (A) and SEM characterizations Mb-conjugated Dynabeads at the concentration of 10 ng/ml (B).
